# What is the role of robotic surgery in ovarian cystectomy with fertility preservation?

**DOI:** 10.1007/s11701-023-01704-w

**Published:** 2023-09-10

**Authors:** Ju Hye Lee, So Yun Park, Kyungah Jeong, Ha Yeoung Yun, Hye Won Chung

**Affiliations:** 1 Department of Obstetrics and Gynecology, College of Medicine, Ewha Womans University Mokdong Hospital, Ewha Womans University, 1071, AnYangCheon-Ro, YangCheon-Gu, Seoul, 07985 Korea; 2 Department of Obstetrics and Gynecology, Ewha Womans University Seoul Hospital, College of Medicine, Ewha Womans University, Seoul, South Korea

**Keywords:** Robotic single-site surgery, Single-port laparoscopic surgery, Anti-Müllerian hormone, Endometriosis

## Abstract

To investigate the role of robotic single-site (RSS) ovarian cystectomy in fertility preservation, which was compared with single-port laparoscopic (SPL) surgery based on AMH changes. We retrospectively analyzed medical records of total 156 patients who underwent SPL (*n* = 72) or RSS (*n* = 84) surgery with the da Vinci^®^ Si or Xi system. The pre/post-operative AMH levels and total diameter of ovarian cysts were measured. In addition to the surgical method, AMH changes were compared according to the laterality, multiplicity, and pathology of ovarian cysts. A comparison of the characteristics of the SPL group and RSS group, revealed that there were no significant differences in the average age, the diameter of the ovarian cyst, and the number of locule. There were also no statistical differences between the pre-operative and post-operative AMH levels and the average surgical time including the docking time in robotic surgery. A comparison based on the surgical methods, revealed that the decrease in post-operative AMH was lower in the RSS group (24.2 ± 35.9%) than in the SPL group (34.9 ± 29.1%) significantly (*p* = 0.044). In patients with endometriosis, the decrease in AMH was greater, than that in patients without endometriosis. A longer operation time, larger ovarian cysts and multi-locular cysts were associated with lower AMH level in both the SPL and RSS groups (Pearson correlation coefficient: – 0.320, *p* = 0.0001, – 0.218, *p* = 0.007, – 0.236, *p* = 0.003, respectively). RSS ovarian cystectomy could be a promising new therapeutic option for fertility preservation in complex cases to avoid an additional side port.

## Introduction

Ovarian tumors are common in reproductive-aged women who visit the gynecologic clinic. In the United States, it has been estimated that as many as 10% of women will undergo surgical removal of an adnexal mass during their lifetime [1]. Particularly, endometriosis is a common gynecological disease that occurs in 5–10% of reproductive-aged women. Its incidence tends to increase gradually with early menarche, delayed pregnancy, and decreased childbirth. Ovarian endometriomas are found in 20% of patients with endometriosis. Although the progestin, dienogest is widely used as a treatment for endometriosis, ovarian cystectomy seems to be the favored modality of most gynecologists, given that complete excision is associated with reduced disease recurrence [2].

Various studies have reported an increased risk of ovarian damage and decreased ovarian reserve after ovarian cystectomy. To preserve ovarian function, suturing for hemostasis after ovarian cystectomy is superior to electro-cauterization with thermal energy [3–5].

Recently, minimally invasive surgery is becoming increasingly popular in the gynecological field owing to a short hospitalization period, rapid recovery, and cosmetic benefits [6]. Most young reproductive-aged women want a fast recovery and less scarring. For this reason, minimally invasive surgery with a single incision is increasingly preferred. In accordance with this trend, single-port laparoscopic (SPL) surgery is being developed as a treatment option. However, SPL surgery has some limitations due to the use of semi-rigid and non-articulating instruments [7, 8].

With the adoption of the da Vinci® system (Intuitive Surgical Inc., Sunnyvale, CA, USA), it has become possible overcome the shortcomings of laparoscopic surgery. Robotic surgery reduces hospital stays and the amount of bleeding during surgery by allowing surgeons to more easily conduct complicated procedures with enhanced visualization and wristed instruments [9].

Anti-Müllerian hormone (AMH) is a dimeric glycoprotein member of the transforming growth factor β-family. [10] It is produced by granulosa cells of small, growing follicles in the ovary. It is also well known for its role in sexual differentiation. Serum AMH level is strongly correlated with the number of growing follicles; thus, AMH has attracted increasing attention as a golden marker of ovarian reserve [11, 12]. Accumulating evidence suggests that AMH is the best currently available measure of ovarian reserve.

**Fig. 1:**
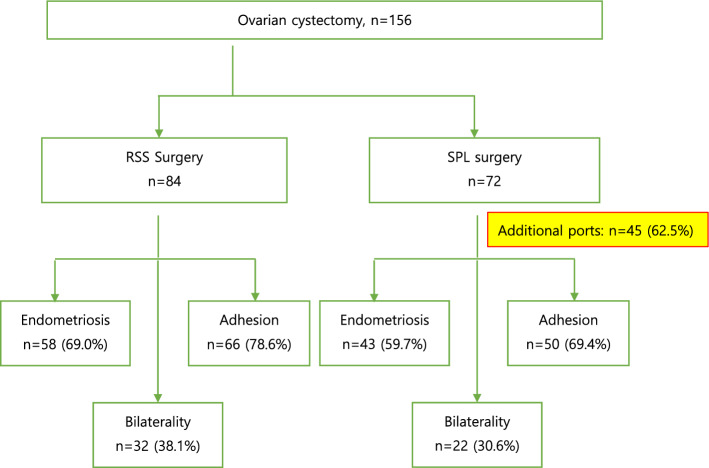
Characteristics of RSS and SPL ovarian cystectomy. *RSS* Robotic single-site; *SPL* Single-port laparoscopic

Therefore, we aimed to investigate the role of robotic single-site (RSS) ovarian cystectomy in fertility preservation, which was compared with SPL surgery based on AMH changes.

## Materials and methods

This study was conducted as a retrospective chart review on 156 patients who were planned for ovarian cystectomy via RSS (*n* = 84) or SPL (*n* = 72) surgery at Ewha Womans University Mokdong and Seoul Hospital from March 2017 to March 2023. All included patients underwent ovarian surgery due to benign gynecologic diseases, which were diagnosed based on the final pathology. Their ages ranged from 16 to 43 years. A total of 72 patients underwent laparoscopic surgery, and 84 patients underwent RSS ovarian cystectomy with the da Vinci Si or Xi system. All operations were performed through the umbilicus with a Glove Port (NELIS, Seoul, Republic of Korea).

Serum samples for AMH measurement were collected from each patient preoperatively and postoperatively. The time of postoperative AMH measurement was determined in months from the time of operation to AMH measurement after the operation. The serum AMH level was measured by electrochemiluminescence immunoassay using the Elecsys AMH Plus reagent (Roche Diagnostics, Mannheim, Germany).

The size of the ovarian cyst was determined based on the sum of the average diameters measured by transvaginal or transrectal sonography before surgery. In addition to the surgical method, AMH changes were compared according to the laterality, multiplicity, and pathology of ovarian cysts. Pearson’s correlation was determined between the percentage of AMH reduction and total operation time, diameter of the ovarian cyst, or number of locule. To evaluate the ovarian reserve after surgery, the change between pre-operative and post-operative serum AMH levels was measured.

Statistical analysis was performed using SPSS version 22 (IBM, Seoul, Republic of Korea). All values with *p* < 0.05 were considered as statistically significant. This study was approved by the Institutional Review Board of Ewha Womans University Mokdong Hospital (IRB No. 2020-04-029).

## Results

All 84 patients in the RSS group underwent ovarian cystectomy through the umbilicus successfully with only one scar. On the other hand, 45 (62.5%) of 72 patients in the SPL group needed one or two additional ports because the SPL approach has some limitations such as the range of motion with rigid instruments. Additionally, in the RSS group, endometriosis, adhesion, and bilaterality accounted for 69%, 78.6%, and 38.1% of patients, respectively compared with those in the SPL group, 59.7%, 69.4%, and 30.6%, respectively (Fig. 1). There were additional complex cases in the RSS group compared with the SPL group.

The clinical characteristics, pre/post-operative AMH levels, number of locule, total diameter of the ovarian cyst, and mean operation time are summarized in Table 1. A comparison of the characteristics of the SPL group and RSS group, revealed that there were no significant differences in the average age (29.0 ± 5.6 in the SPL group vs 28.2 ± 4.5 in the RSS group), the diameter of the ovarian cyst (7.10 ± 3.07 cm in the SPL group vs 7.98 ± 3.70 cm in the RSS group), and the number of locules (1.8 ± 1.3 in the SPL group vs 2.1 ± 1.5 in the RSS group). There were also no statistical differences between the pre-operative and post-operative AMH levels (preoperative AMH 3.59 ± 1.95 ng/mL in the SPL group vs 3.45 ± 1.94 ng/mL in the RSS group, Post-operative AMH 2.53 ± 2.07 ng/mL in the SPL group vs 2.49 ± 1.58 ng/mL in the RSS group) and the average surgical time (95.4 ± 33.2 min in the SPL group vs 89.0 ± 26.7 min in the RSS group) including the docking time in robotic surgery.

**Table 1:** Clinical characteristics of patients

	SPL (*n* = 72)	RSS (*n* = 84)	*p*-value
Age (years)	29.0 ± 5.6	28.2 ± 4.5	NS
Total diameter of ovarian cyst (cm)	7.10 ± 3.07	7.98 ± 3.70	NS
Number of locule	1.8 ± 1.3	2.1 ± 1.5	NS
Duration from operation to post-operative AMH test (month)	5.5 ± 4.1	4.1 ± 7.3	NS
Pre-operative AMH (ng/mL)	3.59 ± 1.95	3.45 ± 1.94	NS
Post-operative AMH (ng/mL)	2.53 ± 2.07	2.49 ± 1.58	NS
Mean operation time (min)	95.4 ± 33.2	89.0 ± 26.7	NS

*SPL* single-port laparoscopic; *RSS* robotic single-site; *AMH* anti-Müllerian hormone

^a^Values are presented as mean ± standard deviation

After surgery, patients with endometriosis received hormone suppression therapy with a GnRH agonist or dienogest, and the measured AMH level ranged from 20 to 30% to 50% lower on average. A comparison of the number of patients who received hormone suppression therapy at the time of post-operative AMH measurement between the two groups, demonstrated that the number of patients in the SPL group (46 of 72 patients; 63.9%) was relatively smaller than that of patients in the RSS group (62 out of 84 patients; 73.8%). The time of post-operative AMH measurement was an average of 5.5 months in the SPL group and 4.1 months in the RSS group after surgery, which showed no significant difference.

AMH levels before and after surgery were compared between the SPL and RSS groups. Data on the percentage of AMH reduction after surgery are shown in Table 2. A comparison based on the surgical methods, revealed that the decrease in post-operative AMH was lower in the RSS group (24.2 ± 35.9%) than in the SPL group (34.9 ± 29.1%), showing a statistically significant difference (*p* = 0.044). In patients with endometriosis, the decrease in AMH was greater (35.9 ± 31.7%), than that in patients without endometriosis (17.8 ± 33.1%), which also showed a statistically significant difference (*p* = 0.001). In addition, AMH reduction was significant in the bilateral group compared with the unilateral group (– 42.7 ± 30.7% in the bilateral group vs – 21.7 ± 32.4% in the unilateral group, *p* = 0.0001). The decrease in AMH was significant in the group with pelvic adhesion compared with the group without adhesion (– 35.6 ± 30.7% in the adhesion group vs – 10.9 ± 33.9% in the non-adhesion group, *p* = 0.0001).

The correlations between the percentage of AMH reduction and various factors were analyzed using Pearson’s correlation coefficient. The correlation coefficient between the total operation time and percentage of AMH reduction indicated a significant negative correlation at – 0.320 (*p* = 0.0001), and the correlation coefficients indicated the significant negative correlation of the diameter of the ovarian cyst and number of locule with the percentage of AMH reduction at – 0.214 (*p* = 0.007) and – 0.236 (*p* = 0.003), respectively (Table 3). In other words, a longer operation time, larger ovarian cysts and multi-locular cysts were associated with lower AMH level in both the SPL and RSS groups.

## Discussion

Limited studies have compared for AMH levels after ovarian cystectomy using a robotic system and a laparoscopic technique. In this study, a comparison between SPL surgery and RSS surgery demonstrated that, unlike the SPL technique, the RSS system was feasible without additional ports, however, there were additional complex cases in the RSS group than in the SPL group due to the disadvantages of SPL surgery such as collisions and clashing of instruments and a limited range of motion [13, 14].

It is well known that endometriomas can result in a greater reduction in AMH level after surgery compared with other benign ovarian cysts such as teratomas of the ovaries due to adhesion. In this study, AMH tended to decrease further when accompanied by endometriosis. In addition, greater AMH reduction in the presence of bilateral ovarian cysts and adhesion suggests that the more complex and difficult the surgery, the more the ovarian parenchyma can be destroyed, and a meticulous surgery should be performed.

**Table 2:** Percentage of AMH reduction after ovarian cystectomy

Type of surgery (%)	SPL (*n* = 72)	RSS (*n* = 84)	*p*-value
	– 34.9 ± 29.1	– 24.2 ± 35.9	0.044
Pathology (%)	Endometriosis (*n* = 98)	Non-endometriosis (*n* = 58)	*p*-value
	– 35.9 ± 31.7	– 17.8 ± 33.1	0.001
Laterality (%)	Bilateral (*n* = 55)	Unilateral (*n* = 101)	*p*-value
	– 42.7 ± 30.7	– 21.7 ± 32.4	0.0001
Adhesion (%)	Adhesion (*n* = 115)	Non-adhesion (*n* = 41)	*p*-value
	– 35.6 ± 30.7	– 10.9 ± 33.9	0.0001

*AMH* anti-Müllerian hormone; *SPL* single-port laparoscopic; *RSS* robotic single-site

^a^Values are presented as mean ± standard deviation

A longer surgery time, a larger ovarian cyst size, and a greater number of locule were negatively correlated with AMH changes. Therefore, in terms of fertility preservation, the RSS system rather than SPL surgery should be used for ovarian cystectomy in more complex cases, such as endometriosis, or larger, multilocular or bilateral cysts with adhesion.

**Table 3:** Correlation with serum AMH changes

Total (*n* = 156)	Pearson’s Correlation Coefficient	*p*-value
Total operation time (min)	– 0.320	0.0001
Diameter of the ovarian cyst (cm)	– 0.214	0.007
Number of locule	– 0.236	0.003

*AMH* anti-Müllerian hormone

Since the initial approval of robotic surgery for gynecologic operation in 2005, its use has been widely adopted, and its application has been expanded. However, the role of robotic surgery in case of benign ovarian tumors including endometriosis remains controversial. Operative times are consistently longer in robotic surgery with no differences in quality of life and fertility outcomes. Berlanda et al. published an article titled “Money for nothing” on the role of robotic assisted laparoscopy in the treatment of endometriosis [15]. Soto et al. reported a multicenter, randomized, controlled trial comparing laparoscopy with robotic surgery for endometriosis in 2017. They concluded that there were no differences in perioperative outcomes between robotic and conventional laparoscopy [16].

However, with the development of robotic systems, RSS surgery has been upgraded with the da Vinci^®^ Xi system. In addition, with the development and release of the 4th generation da Vinci^®^ single-port (SP) system specialized in single-site surgery, robotic surgery has progressed tremendously. All instruments in the da Vinci^®^ SP system have two joints, allowing more delicate movements and the use of strong and diversified instruments rather than conventional unsteady ones, making them considerably more useful. Even if the surgery is complex, the new da Vinci^®^ SP surgical system might be more feasible and safer, than RSS surgery with Si or Xi system. [17]

Ovarian surgery can reduce the ovarian reserve, especially in cases of excessive coagulation for bleeding control. Suturing for hemostasis after ovarian cystectomy is superior to thermal energy in preserving ovarian function [18]. Therefore, it is desirable to preserve ovarian function by avoiding excessive manipulation using energy for hemostasis and suturing rapidly and accurately using a robotic system for easy manipulation. Lee et al. suggested that ovarian function may be better preserved with robotic ovarian cystectomy compared with the laparoscopic approach for bilateral ovarian endometriomas [19].

In the new da Vinci^®^ SP system, the scissors, which were not available in the Si or Xi single-site system, were included to avoid tissue destruction due to electro-cauterization by allowing cold cut and fast suturing as much as possible. In the future, RSS surgery using the da Vinci^®^ SP system could be expected to have a better effect on fertility preservation compared with that of SPL surgery.

Our study has some limitations. First, since it is a retrospective comparison study, more complex surgeries might be selected for the RSS system. Second, the number of patients in the SPL group is slightly lower than that in the RSS group. However, there was no significant difference in characteristics between the two groups.

Although there were additional complex cases and a higher number of hormone suppression therapy patients at the time of post-operative AMH measurement in the RSS group, the decrease in AMH after ovarian cystectomy was lower in the RSS group than in the SPL group. Therefore, the advantages of robotic system for ovarian cystectomy might be supposed in terms of fertility preservation.

In conclusion, RSS ovarian cystectomy could be a promising new therapeutic option for fertility preservation in complex cases to avoid an additional side port. Following the development of a robotic surgery system that could overcome the limitations of laparoscopic surgery for women who need fertility preservation, the application of a robotic surgery system in ovarian cystectomy especially in cases of endometriosis should be considered. Further large-scale prospective cohort studies should be performed to validate the long-term effects of robotic surgery on fertility preservation in benign ovarian cystectomy, including cases of endometriosis, which may be favorable.

## Data Availability

All data supporting the findings of this study are available from corresponding author on reasonable request.
